# Comprehensive Analysis of Cetuximab Critical Quality Attributes: Impact of Handling on Antigen-Antibody Binding

**DOI:** 10.3390/pharmaceutics16091222

**Published:** 2024-09-19

**Authors:** Alicia Torres-García, Anabel Torrente-López, Jesús Hermosilla, Amparo Hernández, Antonio Salmerón-García, José Cabeza, Natalia Navas

**Affiliations:** 1 Fundación para la Investigación Biosanitaria de Andalucía Oriental (FIBAO), Hospital Doctor Olóriz, Avda de Madrid, 15, Pabellón de Consultas Externas 2, 2ª Planta, 18012 Granada, Spain; alicia.torres@genyo.es; 2 Instituto de Investigación Biosanitaria ibs.GRANADA, Avda de Madrid, 15, 18012 Granada, Spain; 3 Analytical Chemistry Department, Science Faculty, Universidad de Granada, Avenida Fuentenueva s/n, 18071 Granada, Spain; anabeltl@ugr.es (A.T.-L.); jesushf@ugr.es (J.H.);; 4 Servicio de Farmacia Hospitalaria, Hospital Universitario San Cecilio, Avenida de la Investigación s/n, 18016 Granada, Spain; antonio.salmeron.sspa@juntadeandalucia.es (A.S.-G.); jose.cabeza.sspa@juntadeandalucia.es (J.C.)

**Keywords:** cetuximab characterization, Erbitux^®^ analysis, stability study, forced degradation, comprehensive analysis, peptide mapping-RP/UHPLC-MS/MS, ELISA

## Abstract

Background/Objectives: Cetuximab, formulated in Erbitux^®^ (5 mg/mL), is a therapeutic monoclonal antibody (mAb) widely used in several cancer treatments. Currently, there is insufficient knowledge about the behavior of cetuximab with regard to the risk associated with its routine handling or unintentional mishandling in hospitals. Forced degradation studies can simulate these conditions and provide insights into the biophysical and biochemical properties of mAbs. Methods: In this study, we conducted a deep physicochemical and functional characterization of the critical quality attributes of cetuximab in control samples and under controlled degraded conditions, including freeze–thaw cycles, heat, agitation, and light exposure. To achieve this purpose, we used a set of proper analytical techniques, including CD, IT-FS, DLS, SE/UHPLC-UV, UHPLC-MS/MS, and ELISA, to check functionality based on antigen–antibody binding. Results: The results revealed that light exposure was the stress stimuli with the greatest impact on the drug product, leading to the formation of non-natural oligomers, fragmentation, and oxidation of methionine residues. Additionally, cetuximab (Erbitux^®^, 5 mg/mL) showed a tendency to aggregate when submitted to 60 °C for 1 h. In terms of functionality, cetuximab (Erbitux^®^, 5 mg/mL) samples were found to be affected when subjected to freeze–thaw cycles, 60 °C (1 h), and when exposed to light (daylight with room temperature excursion and accelerated light exposure). Conclusions: Thus, we suggest that Erbitux^®^ (5 mg/mL) should be shielded from these environmental conditions, as they compromise both the safety and efficacy of the drug product.

## 1. Introduction

Cetuximab is a well-known mAb that is indicated for the treatment of metastatic colorectal cancer, metastatic non-small cell lung cancer, which are two of the most lethal cancers, and squamous cell carcinoma of the head and neck [1]. It is a chimeric IgG1 mAb of 152 kDa composed of four polypeptide chains, including two identical heavy (lambda) chains of 449 amino acids each and two identical light (kappa) chains of 214 amino acids each, linked by disulfide bonds at their hinge region [2,3]. 

Cetuximab binds with high specificity to the extracellular domain III of the epidermal growth factor receptor (EGFR), which is overexpressed in these tumor cells [4,5,6]. Cetuximab competes with its natural ligand, epidermal growth factor (EGF), preventing the dimerization of EGFR and, consequently, inactivating its signal transduction [7]. On the one hand, it blocks cell-cycle progression by inducing a G1 arrest through an increase in the protein levels of p27kip1, an inhibitor of cyclin-dependent kinases. Cetuximab also induces apoptosis in cancer cells by increasing the expression of caspases and altering the Bcl-2/Bax ratio. In addition, the Fc domain of cetuximab binds to CD16a and other Fc receptors to recruit immune mechanisms such as antibody-dependent cellular cytotoxicity. As a result, cell proliferation and tumor growth are thereby downregulated, and angiogenesis and metastasis are reduced [4,5,6].

Cetuximab is the active substance of the innovative medicinal product Erbitux^®^ (5 mg/mL), which is formulated as an intravenous solution for infusion. It is surprising that there is still no biosimilar to this formulation on the market yet, although the first approval was twenty years ago, in 2004. Cetuximab biosimilars are all in phase III development, and none is currently approved for clinical use [8,9]. Erbitux^®^ (5 mg/mL) can be administered as monotherapy or in combination with other therapeutic agents such as irinotecan [1,10]. This biotechnological drug is typically administered in hospitals, where it could be subject to several stress factors during the compounding handling, storage, and administration processes [11], which could make the protein susceptible to degradation due to, i.e., temperature changes, light exposure, shaking, etc. [12,13]. Degradation pathways could involve physical degradation of the secondary and tertiary structure caused by denaturation, aggregation, or fragmentation, as well as chemical degradation inducing post-translational modifications (PTMs), i.e., isomerization, deamidation, oxidation, etc. These modifications alter the quality of mAbs and can have a significant impact not only on their therapeutic efficacy by limiting their activity but also on their safety through an increase in immunogenicity. For this reason, mAbs degradation must be detected before administration to patients by hospital pharmacists to ensure the quality, efficacy, and safety of the formulation [14,15]. Understanding the impact of deviations from proper handling would allow for the identification of the common situations in the daily use of this medication that promote and lead to degradation.

Stability studies based on controlled degradation become the way to provide valuable information about physicochemical changes and degradation pathways that may occur in mAbs. These studies are designed to expose the therapeutic protein to defined and controlled experimental stress conditions. The guidelines of the International Council for Harmonization of Technical Requirements for Pharmaceuticals for Human Use (ICH) do not provide details of the practical issues of stress testing in biopharmaceuticals during stability studies, except for photostability testing [16,17,18]. However, the available bibliography provides information about stability studies involving mAbs and other proteinaceous drugs (Fc-fusion proteins, peptides, etc.). These studies aim to simulate the stress stimuli to which the drug may be subjected during hospital handling, which typically include high temperatures, freeze–thaw cycles, agitation, or light exposure, in order to study the effects, which may cause a lack of quality, safety, and efficacy using appropriate analytical tools [12,13,19].

To evaluate mAbs stability, critical quality attributes (CQAs) have to be checked. They are defined by the ICH guideline Q8 (R2) on pharmaceutical development as “physical, chemical, biological or microbiological properties and characteristics that should be within an appropriate limit, range or distribution to ensure product quality”. Although not all modifications are relevant to quality control, certain variations can be considered CQAs, as they influence product quality. The complexity of the analysis of the CQAs resides in the requirement to employ high-precision and high-sensitivity techniques for the very different physicochemical properties, in addition to the functionality, capability of discerning atomic-level chemical modifications, sizing variations with maximum precision and accuracy, and tiny changes in drug avidity, which enables the identification of which chemical or structural variations are likely to exert an impact on mAb quality. 

Thus, in this study, a variety of complementary techniques were used to achieve a comprehensive characterization of cetuximab, including (i) far-ultraviolet circular dichroism (CD) spectroscopy for the evaluation of the secondary structure; (ii) intrinsic tryptophan fluorescence spectroscopy (IT-FS) for the analysis of the tertiary structure; (iii) dynamic light scattering (DLS) to study the particulate in the solutions of the samples; (iv) SE/UHPLC-UV to analyze aggregation/fragmentation alterations; (v) peptide mapping-((RP)UHPLC-(Orbitrap)MS/MS) for the detection and quantification of chemical modifications in the primary structure; and (vi) ELISA to evaluate the biological activity. This comprehensive analysis strategy has proven to be successful in previous studies that also focused on the impact of the handling conditions on several mAbs [12,20,21], since it can provide an in-depth understanding of the intrinsic stability of the particular mAb studied, as well as identify key degradation mechanisms and their impact on quality, efficacy, and safety. Thus, knowledge of the potential risks associated with their routine handling allows the hospital pharmacist to manage accidental exposure to unrecommended conditions and ensure that the drug meets the quality requirements before patient administration [14,22].

## 2. Materials and Methods

### 2.1. Chemicals and Reagents

Erbitux^®^ (5.0 mg/mL, Merck Europe B.V.; Amsterdam, The Netherlands) was kindly supplied by the Pharmacy Unit of the University Hospital San Cecilio (Granada, Spain). It is formulated as an intravenous solution for infusion, whose complete formulation includes sodium chloride, glycine, polysorbate 80, citric acid monohydrate, sodium hydroxide, and water for injections [1]. Several batches were analyzed during the study, including G01EM9, G01D53, G01DMN, G01GD1, G0199R, G01B92, and G01M08.

Reverse-osmosis-quality water was purified with a Mili-Q station from Merck Millipore (Merk KGaA, Darmstadt, Germany). Anhydrous disodium hydrogen phosphate and monohydrate monobasic sodium phosphate were supplied by Panreac (Barcelona, Spain) and Sigma-Aldrich (Merk KGaA, Darmstadt, Germany), respectively. Formic acid (FA) was obtained from Thermo Fisher Scientific (Waltham, MA, USA) and acetonitrile (ACN) from VWR International Eurolab S.L (Barcelona, Spain), both of LC/MS quality. For enzymatic digestion, a SMART Digest Soluble Trypsin Kit from Thermo Fisher Scientific (Waltham, MA, USA) was used. Dithiothreitol (DTT) and iodoacetamide (IAA) were obtained from Sigma-Aldrich (Merk KGaA, Darmstadt, Germany), while trifluoroacetic acid (TFA) was obtained from Scharlab S.L (Barcelona, Spain). The recombinant human EGFR was obtained from Abcam (Cambridge, UK) and goat anti-human IgG1 Fc antibody-HRP conjugated from Merck Millipore (Merk KGaA, Darmstadt, Germany). O-Phenylenediamine Dihydrochloride (OPD) and Tween^®^ 20 were supplied by Sigma Aldrich (Merk KGaA, Darmstadt, Germany). Sulfuric acid, sodium chloride, sodium hydroxide (pure for analysis), sodium carbonate, and monohydrogen sodium phosphate were supplied by Panreac (Barcelona, Spain). Guanidine hydrochloride was supplied by Sigma-Aldrich (Merk KGaA, Darmstadt, Alemania). Skimmed milk powder was obtained from La Asturiana (Siero, Spain).

### 2.2. Forced Degradation Conditions

The forced degradation conditions were selected according to the accidental exposures to which the drug may be subjected during the different stages of handling in the hospital pharmacy. One hundred µL of Erbitux^®^ (5 mg/mL) were subjected to the following stress conditions: (i) heat exposure at 40 °C and 60 °C for 1 h in an Eppendorf ThermoMixer^®^ C thermomixer (Eppendorf, Hamburg, Germany); (ii) freeze–thaw cycles, with one (1 FTC) and three cycles (3 FTC) from room temperature to −20 °C using a convectional freezer; (iii) exposure to daylight for 24 h with thermal excursion from 10 to 31 °C; (iv) exposure to accelerated light irradiation (250 W/m^2^) for 24 h in an aging chamber (Solarbox 3000e RH, Cofomegra, Milan, Italy) following ICH Q1B (Photostability Testing of New Drug Substances and Products); and (v) shaking at 300 rpm for 24 h protected from light in a GFL-3006 horizontal shaker (GFL, Burgwedel, Germany). Erbitux^®^ (5 mg/mL) kept under manufacturer recommendations (4 °C) was used as a control sample, while a denatured sample in GndHCl 8M (final cetuximab concentration of 1.25 mg/mL) was used as positive control of degradation to check the secondary and tertiary structures. All conditions were analyzed immediately after the stress exposure and simultaneously with the control samples (kept under manufacturer recommendations (4 °C), not subjected to stress, and analyzed right after opening the vials), in order to avoid any other degradation that might interfere with the results.

### 2.3. Physicochemical Analytical Methods

#### 2.3.1. Visual Inspection

All the samples were inspected by the naked eye prior to experimentation to check their aspect and look for evidence of turbidity, color changes, or aggregation. These inspections were performed by two people (4-eye principle) using a black-and-white background.

#### 2.3.2. Far Ultraviolet (UV) Circular Dichroism Spectroscopy

Cetuximab’s secondary structures were evaluated through CD spectroscopy within the far-UV range (190–260 nm). The experimental setup mirrored conditions similar to those described in [12], utilizing a JASCO J-815 spectropolarimeter (JASCO, Tokyo, Japan) with a Peltier system for temperature regulation, consistently maintained at 20 °C during all measurements. Both control and stressed samples (5 mg/mL) were diluted in water to achieve a cetuximab concentration of 0.2 mg/mL. Spectra were recorded from 260 to 190 nm at 0.2 nm intervals, employing a scan speed of 50 nm/min. Each spectrum resulted from eight accumulations, recorded with a 2 nm bandwidth. The analyses utilized an absorption QS quartz macro cell 100-1-P-40 (Hellma Analytics, Munich, Germany) with a 1 mm optical path length. Prior to spectrum registration, a blank measurement was taken and subtracted. Spectra Manager software version 2 was employed for applying Savitzky–Golay smoothing to all spectra.

Following stress testing, various spectral characteristics were monitored, including the wavelength (nm) at ellipticity = 0, the negative maximum (nm), and the broad shoulder (nm). Additionally, to estimate the secondary structure content (%), the Dichroweb server was employed, selecting the algorithm and data set with the lowest normalized root-mean-square deviation (NRMSD) fit parameter [23].

#### 2.3.3. Intrinsic Tryptophan Fluorescence Spectroscopy (IT-FS)

Intrinsic tryptophan fluorescence spectroscopy (IT-FS) was employed to analyze the tertiary structure of cetuximab in both the stressed and control samples (5 mg/mL). A Cary Eclipse spectrofluorometer (Agilent, Santa Clara, CA, USA) was used to perform the IT-F measurements. Fluorescence emission spectra, obtained by selectively exciting tryptophan residues at 298 nm, were recorded in the range of 300 nm to 450 nm. These recordings took place at room temperature with the excitation and emission slits set at 5 nm each, and a total of 10 spectral accumulations were collected for all measurements at a scan speed of 600 nm/min.

The spectral center of mass (C.M.) was treated as a mathematical depiction for each spectrum and was computed employing Equation (1) over the range of 300 nm to 450 nm:

(1) $$C.M.=\frac{\sum_{i}^{n}\lambda i\times Fi}{\sum_{i}^{n}Fi}$$

where *λi* is the wavelength associated with its fluorescence intensity *Fi*.

#### 2.3.4. Dynamic Light Scattering (DLS)

The particle size distribution of cetuximab (Erbitux^®^, 5 mg/mL) in both the control and stressed samples, spanning from 1 nm to 10 µm, was characterized through dynamic light scattering (DLS). Photon correlation spectroscopy was employed to assess the population’s mean hydrodynamic diameter (Dh) and the polydispersity index (PDI). This analysis utilized a Zetasizer Nano ZS-90 (Malvern Panalytical, Malvern, UK) equipped with a backscattered light detector operating at 90° and 25 °C. The temperature for this study was set at 20 °C. Each measurement had an acquisition time of 5 s per read, with 50 reads recorded per measurement. The results were computed using cumulants analysis with Zetasizer Software version 8.01 (Malvern Panalytical, Malvern, UK). In all measurements, a 1 cm spectrophotometry disposable cuvette was utilized. The data were processed using the protein analysis model to obtain the size distribution of all the analyzed samples.

#### 2.3.5. Size-Exclusion Ultra-High-Performance Liquid Chromatography with UV Detection (SE/UHPLC-UV)

The analysis was performed using a Dionex UltiMate 3000 chromatograph (Thermo Scientific, Waltham, MA, USA) equipped with two ternary pumps, a degasser, an autosampler, a thermostatic column compartment, and a multiple-wavelength detector (MWD-3000 Vis-UV detector). The size exclusion analysis separation was carried out in an AdvanceBio SEC column 300 Ȧ, 2.7 µm, 4.6 × 300 mm (Agilent Technologies Inc., Santa Clara, CA, USA). The column was previously calibrated using a calibration kit (AdvanceBio SEC 300A Protein Standard, Agilent Technologies Inc., Santa Clara, CA, USA) that contains 5 proteins, including thyroglobulin (670 kDa), γ-globulin (150 kDa), ovalbumin (45 kDa), myoglobin (17 kDa), and angiotensin II (1 kDa) (Table S1 and Figure S1).

Eight µL of the drug product were analyzed in isocratic mode using 150 mM of phosphate buffer pH 7.0 for 18 min. The flow rate was set at 0.3 mL/min. UV chromatograms were registered at three wavelengths, namely 214 nm, 220 nm, and 280 nm.

#### 2.3.6. Enzymatic Sample Digestion

Enzymatic digestion was performed using a SMART Digest™ Soluble Trypsin Kit from Thermo Fisher Scientific (Waltham, MA, USA). The disulfide bond reductions of both the control and stressed samples (50 µL of cetuximab diluted at 2 mg/mL) were carried out using 10 µL of 100 mM DTT incubated for 30 min at 57 °C and alkylated with 25 µL of 100 mM IAA in darkness for 30 min at room temperature. Then, all of the samples were digested with 5 µL of soluble trypsin (SMART Digest™) for 45 min at 70 °C and shaking at 1400 rpm, with a post-reaction cooling down to room temperature. After that, 15.45 µL 100 mM DTT (to eliminate the excess of IAA) and 15.61 µL of TFA 10% in water (to quench the digestion assuring a pH ˂ 3) were added. Finally, it was centrifuged at 13,000 rpm for 10 min. The supernatant was transferred to an insert that was kept in HPLC amber vials to be analyzed by LC-MS/MS.

#### 2.3.7. Peptide Mapping Ultra-High-Performance Liquid Chromatography Coupled with a Heated Electro-Spray Ionization Tandem Mass Spectrometry (UHPLC-HESI(Orbitrap)MS/MS)

The chromatographic system used for the separation, identification, and quantification of the resulting peptides from enzymatic digestion was the same as described in Section 2.3.5, coupled in line with a Q-Exactive Plus mass spectrometer (Thermo Scientific, USA).

Based on the available literature [24], the analysis was performed by injecting 5 µL of the peptide solution that resulted from the enzymatic cleavage. It was applied to a binary gradient of 0.1% (*v*/*v*) formic acid in water (mobile phase A) and 0.1% (*v*/*v*) formic acid in acetonitrile (mobile phase B). The gradient conditions were set as follows: 2% B increased to 40% B in 45 min, with a further increase to 80% B in 1 min. The gradient was kept at 80% B for 4 min and, then, shifted to 2% B in 0.5 min. Lastly, 2% B was kept for 5 min for column reconditioning. The flow rate was 0.3 mL/min. The separation of the peptides was developed in an Acclaim^TM^ RSLC 120 C18, 2.2 µm, 2.1 × 250 mm column (Thermo Fisher Scientific, Waltham, MA, USA), and the temperature was kept at 25 °C. The ionization was performed in positive mode using a heated electro-spray ionization (HESI) source. The subsequent HESI settings were as follows: spray voltage 3.8 kV, sheath gas flow rate 40 AU, auxiliary gas flow rate 10 AU, and capillary temperature 320 °C. The MS method consisted of full positive polarity MS scans at 70,000 resolution setting (at *m*/*z* 200) with the mass range set to 200–2000 *m*/*z* and an AGC target value of 3.0 × 10^6^, with a maximum injection time of 100 ms and 1 microscan. The in-source CID was set to 0 eV. The MS2 settings were as follows: a resolution setting of 17,500 (at *m*/*z* 200), AGC target value of 1.0 × 10^5^, isolation window set to 2.0 *m*/*z,* and a maximum IT of 200 ms.

### 2.4. Functional-Based Method: Enzyme-Linked Immunosorbent Assay (ELISA)

An indirect non-competitive ELISA method based on the available bibliography was adapted to study cetuximab’s biological activity [3,12,20]. The plate was sensitized with 100 µL/well of 2 µg/mL recombinant human EGFR dissolved in 0.1M carbonate buffer solution pH 9.6 and incubated overnight (18 h) at 4 °C. After that, the plate was washed automatically with an Intelispeed Washer IW-8 (Biosan, Riga, Latvia) for four times with 200 µL/well of PBS-Tween 20 0.3% (*v*/*v*) solution at pH 7.4. Then, the plate was treated with 200 µL/well of the blocking buffer (PBS-Tween 20 pH 7.4, containing skimmed milk 2% (*w*/*v*)) for 2h at 37 °C to eliminate nonspecific adsorptions. After this time, the plate was washed again following the indications exposed previously. For the calibration model, 100 µL of Erbitux^®^ (5 mg/mL) at 0.001, 0.005, 0.01, 0.05, 0.1, 1, 5, and 25 µg/mL diluted in 0.1 M carbonate buffer of pH 9.6 were analyzed in triplicate. The samples were incubated for 45 min at 37 °C and washed as indicated before. One hundred µL/well of 1:5000 diluted anti-human IgG1-HRP in 0.1 M carbonate buffer solution pH 9.6 were added and incubated for 30 min at 37 °C. After washing again, 100 µL of the substrate solution (OPD) was added to each well., which was incubated for 20 min at room temperature in darkness. Finally, the reaction was stopped by adding 50 µL/well of 1 M sulfuric acid solution. The plate was analyzed with NanoQuant Infinite 200 Pro (Tecan Trading AG, Männedorf, Switzerland), detecting the absorbance at 450 nm and 620 nm. The analytical signal was established as the difference between both absorbances.

The precision and accuracy of the method were evaluated according to Q2(R2) ICH guidelines [25]. Then, three concentrations were used for checking the precision and accuracy, considering the optimal 0.01, 0.05, and 0.1 µg/mL according to the following criterion: the concentrations shall be placed within an intermediate range of the calibration curve where the slope is maximum. Precision was studied as repeatability (intraday precision) and intermediate precision (interday precision). Repeatability involved the analysis of 10 samples prepared within the same day and under the same experimental conditions (at target concentrations). Intermediate precision entailed the analysis of 3 samples, also at each of those three concentrations, repeated on three different days under the same experimental conditions. The results are presented as the relative standard deviation (RSD%) of each concentration tested. Accuracy was evaluated through the analysis of 5 samples at the three mentioned concentrations, and the results were expressed as the mean recovery (R%) for each one.

This protocol was used for the stability study, using the target concentrations 0.01, 0.05, and 0.1 µg/mL, as indicated previously. Both the control and stressed samples were analyzed with the aim of comparing their functionalities, providing statistically significant results by using the Student’s *t* test. The functionality for each degraded condition checked was determined as the remaining biological activity percentage (RBA%). The results of the statistical tests and the value of the RBA% were selected as criteria to determine significant functionality loss, including (1) a *p*-value ≤ 0.05 in the statistical test at a minimum of two data points and (2) an RBA% value ˂ 90%.

### 2.5. Data Processing

The processing of the CD spectra was performed using Spectra Manager software version 2, while the estimation of the secondary structure content (%) was conducted in the Dichroweb server [23]. DLS data were analyzed using Zetasizer Software version 8.01 (Malvern Panalytical, Malvern, UK). SE/UHPLC-UV data analysis was carried out using Xcalibur QualBrowser 4.0 (Thermo Fisher Scientific, Waltham, MA, USA). The peptide mapping data processing, identification, and quantitation were performed on BioPharma Finder ver. 5.1 software (Thermo Fisher Scientific, Waltham, MA, USA). Microsoft Excel (Microsoft 365, Microsoft, Redmond, WA, USA) and GraphPad Prism ver. 8 (Dotmatics, Woburn, MA, USA) software were used to obtain the calibration curves, process the statistical data, and represent the data graphically.

## 3. Results

### 3.1. Visual Inspection

Cetuximab is a liquid, transparent, and without visible particulate matter. All samples remained unchanged after stress stimuli exposure. There were no precipitates, turbidity, or particulate matter visible to the naked eye in any of the samples.

### 3.2. Far Ultraviolet (UV) Circular Dichroism Spectroscopy

**Figure 1:**
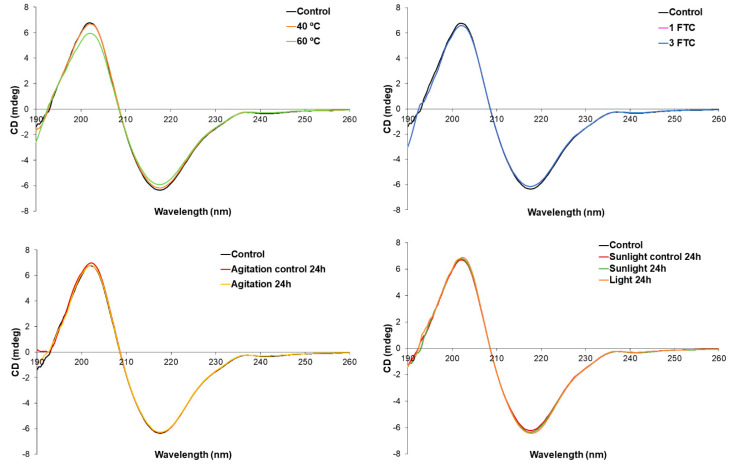
Far-UV CD spectra of cetuximab (5 mg/mL) stressed and control samples.

Figure 1 depicts the CD spectra of the stressed samples in comparison to their corresponding controls. Table 1 presents the primary spectral characteristics, including the wavelength (nm) at ellipticity 0, the negative peak, and the broad shoulder. Additionally, Table 2 provides the estimation of secondary structure content in percentage.

The CD spectrum of the cetuximab control exhibits a wavelength at ellipticity 0 of 208.8 ± 0.1 nm, a negative peak at 217.8 ± 0.1 nm, and a broad shoulder at 229.4 ± 0.1 nm. This spectrum reflects a predominant β-sheet (42%) and unordered (33%) structure, with minimal turns (22%) and α-helix (3%) components (Table 2). These parameters remained consistent across most stressed samples, indicating the preservation of pembrolizumab’s native secondary structure under various stress conditions.

At first glance, slight differences in the CD spectra can be observed in the sample subjected to 60 °C when compared to the control sample (Figure 1). However, upon comparing the parameters collected in Table 1, there are no significant differences regarding the control sample. The estimation of secondary structure content revealed a minor rise in α-helix content and a reduction in β-sheet content for the sample subjected to 60 °C, although again, it is not a significant change (Table 2). The rest of the stressed samples showed similar results to the control sample, concluding that no changes in the protein’s secondary structure occurred for any of the samples subjected to the different stressful conditions.

### 3.3. Intrinsic Tryptophan Fluorescence Spectroscopy (IT-FS)

**Figure 2:**
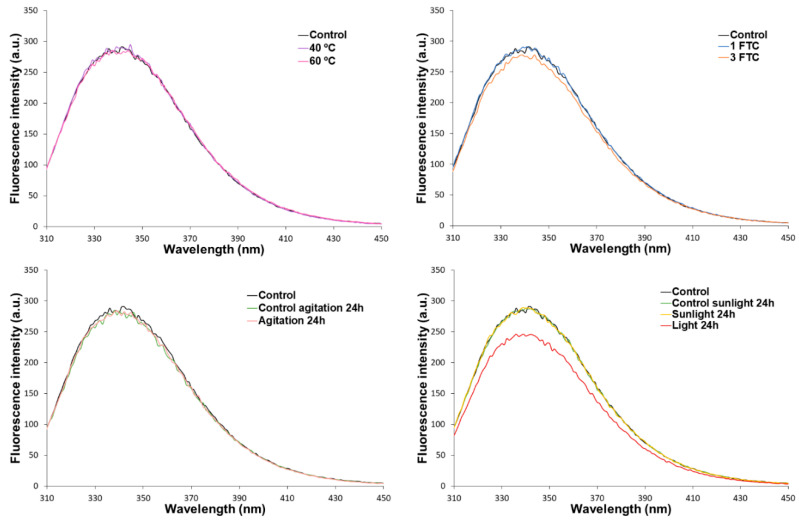
Intrinsic tryptophan fluorescence emission spectra of cetuximab (Erbitux^®^, 5 mg/mL) stressed and control samples.

The IT-FS and C.M. calculation of the obtained fluorescence spectra were selected for tertiary structure analysis. The results from IT-FS are presented in Figure 2 and Table 3. Figure 2 displays the fluorescence emission spectra of both the stressed and control cetuximab samples, while Table 3 presents the C.M. calculated using Equation (1). The cetuximab control (Erbitux^®^, 5 mg/mL) exhibits a C.M. of 351 nm corresponding to an emission maximum of approximately 340 nm, indicating that the Trp residues are situated in the macromolecule’s core, largely shielded from the solvent [26]. None of the stress conditions evaluated in this study affected this parameter, as the C.M. value remained constant for all of the stress conditions tested (Table 3). It is noteworthy that the fluorescence signal decreased significantly after 24 h of accelerated light stress (Figure 2). This decrease could be attributed to a reduction in the fluorescence signal of the tryptophan residues of cetuximab, likely due to degradation, as light stress is a well-known oxidizing agent [11]. Tryptophan residues may undergo oxidation, resulting in a decrease in the intensity of the IT-FS spectrum. This result is similar to that previously obtained with a similar protein (nivolumab) within our research group [12].

### 3.4. Dynamic Light Scattering (DLS)

The main results obtained by DLS are shown in Table 4, i.e., the average HD of the populations detected and the PDI for cetuximab (Erbitux^®^, 5 mg/mL) control and stressed samples.

The DLS control sample (Erbitux^®^, 5 mg/mL) is characterized by a unique population (Figure S2a), with an average HD of 12.8 ± 2.9 nm and a PDI value of 0.26. Therefore, this population was mainly monodispersed and associated with cetuximab monomers in solution. After stressing the samples (Erbitux^®^, 5 mg/mL), the HD and the PDI values of most of the conditions tested remained similar to the control sample, except for the 60 °C experiment. In this case, the HD and its standard deviation (SD) slightly increased from 12.8 ± 2.9 nm to 13.2 ± 5.0 nm. This occurred similarly to the PDI value, where an increase from 0.26 to 0.33 was observed. This can be explained by a partial denaturation and starting aggregation of cetuximab. Moreover, Figure S2b shows an increased width of the peak base, corroborating the presence of new particles of higher size.

### 3.5. Size-Exclusion Ultra-High-Performance Liquid Chromatography with UV Detection (SE/UHPLC-UV)

Control and stressed samples were analyzed by SE/UHPLC-UV in order to compare their chromatographic profiles and detect any fragmentation or aggregation of the protein.

**Figure 3:**
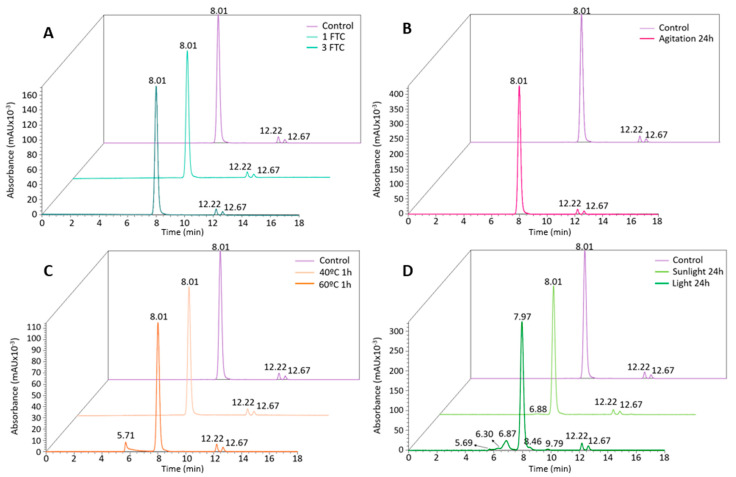
Representative chromatograms SE/UHPLC-UV cetuximab samples. (**A**) Freeze-thaw cycles stress, (**B**) agitation stress, (**C**) temperature stress, and (**D**) light stress. The retention times indicated in the chromatograms are the means of three replicates.

In the control chromatograms, three peaks were detected at the following retention times: 8.01 min (95.6% of the total area), 12.20 min (2.6%), and 12.67 min (1.8%), as depicted in Figure 3. The molecular weights of these three compounds were estimated, resulting in 156.36, 4.09, and 2.74 kDa, respectively. According to the EMA, the molecular weight of cetuximab is approximately 152 kDa, including glycosylation [27]. Thus, the retention time corresponding to 8.01 min was assigned to the natural monomer. There were no dimers nor higher orders of aggregation detected in the control samples. As exposed in our prior studies, the control sample stored under the manufacturer-specified conditions should not have experienced degradation. Hence, it is assumed that the peaks observed at 12.20 and 12.67 min are intrinsic to the sample, potentially corresponding to excipients present in the drug [11,13,28]. Considering that neither the retention time nor the relative abundance (RA%) underwent alterations for these two peaks when subjected to the forced degradation process, as in our previously cited published works, it can be assumed that they are not related to proteinaceous material. Then, given that the primary focus of the present study is the protein drug Erbitux^®^ (5 mg/mL), we examined changes in the chromatographic profile with respect to the monomer. Therefore, we established the peak detected at 8.01 min as representing 100% relative abundance of the protein fraction.

Regarding the stress conditions, no changes were detected with respect to the SE chromatographic profile of the control samples when the samples were submitted to FTC, agitation, or 40 °C (Figure 3A–C). However, samples exposed to 60 °C for 1 h showed aggregation. It was detected a peak at 5.70 min (8.06% of the protein fraction), which was assigned to oligomer formation (Figure 3C). These findings are consistent with the prior studies conducted by our research group, where it was observed that subjecting Erbitux^®^ (5 mg/mL) to a temperature of 50 °C for 24 h resulted in the formation of aggregates [28]. However, in this previous study, disruptions in the chain were also observed, which were not detected in the present experience, probably because of the differences in stress exposure duration between both studies (1 here instead of 24 h previously). Degradation promoted by light irradiation (Figure 3D) in a UV aging chamber gave rise to a degraded pattern that involves both aggregation (three HMWS) and fragmentation (two LMWS) species. After the exposition, 10.71% of the monomers (7.97 min elution time) were transformed into trimers (6.87 min). Furthermore, 0.54% and 2.06% of the monomers displayed an aggregation pattern that was assigned to an emerging oligomer formation of around eight and five units of monomers at 5.68 min and 6.30 min, respectively. These results are consistent with our previous studies, where a clear aggregation pattern was also detected during the initial 24 h of exposure to light, reaching an aggregate relative abundance at the final time point (17 days) of 85.5% of the protein fraction [28]. Also, two fragments were detected at higher retention times, i.e., (8.46 min and 9.79 min), representing 1.96% and 0.79% of the protein fraction. By the estimated molecular weights, a peak at 8.46 min could be assigned to the Fab fragment and a peak at 9.79 to the Fc fragment. It is worth indicating that, although the major degradation pathway induced by light exposure is aggregation, fragmentation around the hinge region is also commonly observed, such as is here proposed by the results obtained. Similar to accelerated light irradiation, in the samples exposed to daylight, the trimer was detected (6.88 min but at a lower percentage and 0.80% of the protein fraction) (Figure 3D). No other degradation pathways were detected in these samples. These results are summarized in Table 5, which includes the standard deviation of the retention times and the relative abundances.

### 3.6. Peptide Mapping Ultra-High-Performance Liquid Chromatography Coupled with Tandem Mass Spectrometry (RP/UHPLC-(Orbitrap)MS/MS)

**Figure 4:**
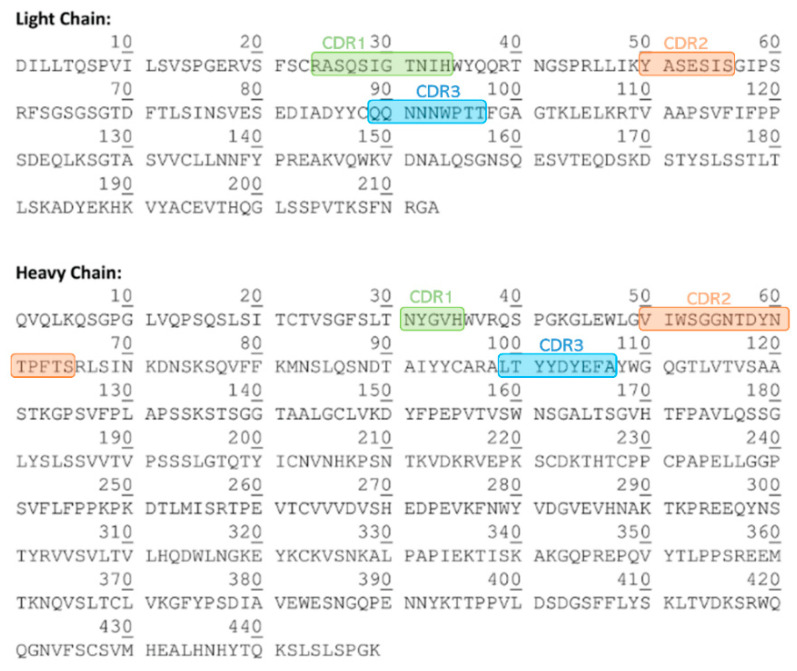
Cetuximab primary sequence. Regions highlighted in green, orange, and blue correspond to CDR1, CDR2, and CDR3, respectively. Adapted from Ayoub D et al. [34].

Post-translational modifications in the primary structure have a crucial role in the efficacy and safety of mAbs [29]. These PTMs frequently occur on therapeutic mAbs, resulting in significant molecular heterogeneity and potential effects on the biological function of the protein. According to the literature, the most prevalent PTMs were specifically analyzed, including the deamidation of asparagine (Asn, N) and glutamine (Gln, Q), oxidation of methionine (Met, M) and tryptophan (Trp, W), isomerization of aspartate (Asp, D), N-glycosylation, formation of N-terminal pyroglutamate, and processing of C-terminal lysine [30]. The effect of PTMs is largely dependent on the specific location of the affected amino acid residues, so changes within the CDR of an antibody may have a great impact on the binding affinity to target antigens [30,31,32]. The complete sequence of cetuximab and the location of CDRs in the primary structure is represented in Figure 4.

Cetuximab enzyme digestion provided 99.1% and 100% sequence coverage for the heavy and light chains, respectively, in all samples with a confidence of 0.90. This is shown in Figure S3. The total results were filtered within 5 ppm (absolute value), reporting a total of 66 PTMs in the cetuximab primary structure. Of these, only 13 PTMs were detected above 5% of relative abundance. As reported in the available bibliography, a certain percentage of PTMs might arise during the enzymatic digestion process, so values below 5% were not considered to study CQAs [33].

Although a total of 19 deamidations were reported, none of them reached a relative abundance of 5%. As previously mentioned, these deamidations may have been induced by the sample treatment, as they are susceptible to pH changes [35]. Overall, the results indicated that deamidation appeared to be unaffected by the stress conditions applied.

**Figure 5:**
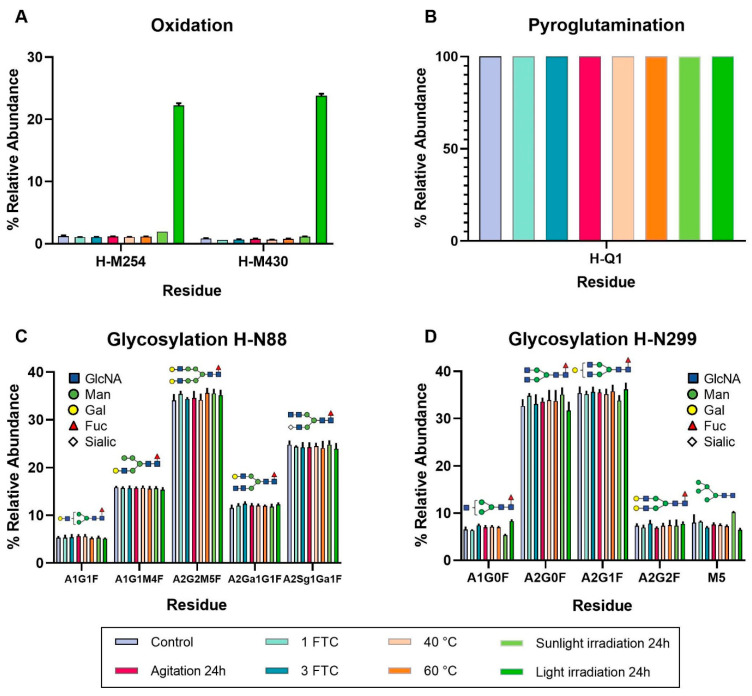
Relative abundance of most important PTMs identified in cetuximab. (**A**) Oxidations, (**B**) pyroglutamination, (**C**) glycosylation located on H-N88 residue, and (**D**) glycosylation located on H-N299 residue. Error bars depict standard deviation (n = 3).

Two oxidations were reported with a relative abundance value exceeding 5%: H-M254 and H-M430. Figure 5A shows that, in both cases, their relative abundances showed a slight oxidative increase when cetuximab was exposed to daylight irradiation, whereas exposure to accelerated light irradiation through the UV aging chamber resulted in a drastic increase compared to the control, reaching relative abundance values of 22.23% and 23.78%, respectively. It is important to note that, although the levels of light-induced oxidation in H-W52 and H-W94 residues only increased up to 2.55% and 1.67%, respectively, both residues belong to the heavy-chain CDR sequence. The impact of oxidation greatly depends on the location of the oxidized residues. Oxidation of residues located within the CDR of an antibody may negatively affect its binding to target antigens, while oxidation of methionine and tryptophan side chains has been shown to induce conformational changes, affect antibody binding to Fc receptors and antigens, and influence mAb stability and half-life. Immunogenicity may also be increased by oxidation of methionine residues [32]. Therefore, the oxidation of H-M254, H-M430, H-W52, and H-W94 residues represents critical modifications for quality control. Although the oxidation of H-W52 and H-W94 residues has the potential influence to alter the antigen-binding capabilities of cetuximab, impairing its therapeutic activity, the relative abundance was too low to significantly impact the EGFR-cetuximab binding. This is not the case for the oxidations in H-M254 and H-M430 residues, which can significantly impact cetuximab’s stability, half-life, and immunogenicity.

Interestingly, the loss of ammonia in the H-Q1 residue was detected in all cetuximab samples, both control and stressed, with a relative abundance of 100% (Figure 5B). We suggest that this ammonia loss detected by the BioPharma Finder 5.1 software leads to the formation of an N-terminal pyroglutamate as glutamine conversion to terminal pyroglutamate involves an ammonia loss [36]. This finding indicates that pyroglutamination is an inherent modification of cetuximab and does not occur as a post-translational modification under stress conditions. Neither isomerization nor C-terminal lysine clipping was reported as a significant post-translational modification in any of the samples analyzed.

N-glycosylation is a highly significant PTM in antibodies, contributing to mAb activity, stability, solubility, immunogenicity, and half-life. Therefore, it is crucial to characterize the N-glycan profile to ensure product quality and control batch-to-batch variations [29]. According to the literature, two different N-glycosylation points in cetuximab, located in H-N88 and H-N299 residues, were reported. It is important to note that all mAbs are N-glycosylated at the conserved asparagine residue (H-N299) in the CH2 domain of the Fc region. However, approximately 15–20% of mAbs are also N-glycosylated in the variable Fab region of either the heavy or the light chain [29,37], providing a high degree of structural complexity [35]. It is worth noting that N-glycans are robust and require highly aggressive stress levels to alter their structure [29,37]. Our results confirmed this robustness, as they remained unaffected even under the most aggressive stresses.

In the H-N88 residue, the most prevalent glycoform was A2G1M4F, accounting for an approximate relative abundance of 35% across all tested conditions. It was followed by A2Sg1Ga1F (24% relative abundance), A1G1M4F (15% relative abundance), A1Ga1G1F (12% relative abundance), and A1G1F (5% relative abundance) (see Figure 5C). Glycoforms A3Ga1G2 and A2Sg1G1F were also detected, although in a lower relative abundance (less than 5%).

**Figure 6:**
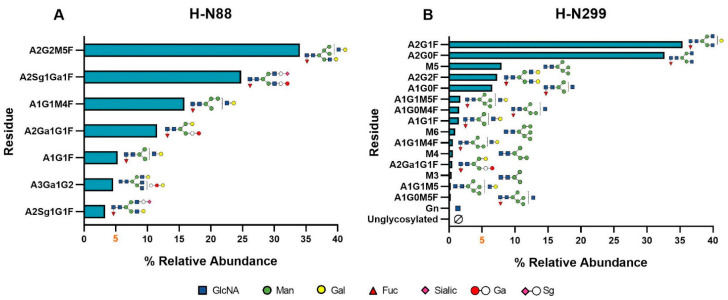
Comprehensive glycan profile of cetuximab. The various isoforms are arranged in decreasing order of relative abundance (%) based on the control sample both in (**A**) H-N88 residue and (**B**) H-N299 residue.

Regarding glycoforms detected in the H-N299 residue, they are reported in descending order of relative abundance, as follows: A2G1F (35%), A2G0F (33%), M5 (7%), A2G2F (7%), and A1G0F (6%) (see Figure 5D). Several other glycan structures were detected with a relative abundance below 5%. Figure 6 illustrates the comprehensive profile of glycans present in the primary structure of cetuximab, arranged in descending order of relative abundance.

These findings are in agreement with previous studies, such as Qian et al. (2007), who reported that cetuximab Fc glycans exhibit lower complexity, predominantly characterized by biantennary fucosylated and high-mannose structures. In contrast, the author also reported that Fab glycans are more complex because they are more extensively galactosylated and contain higher levels of sialic acid [38]. Moreover, Fussl et al. (2020) conducted an exhaustive analysis of the cetuximab glycan profile, identifying prevalent and minority glycoforms in both the Fab and Fc regions. In Fab regions, A2Ga2F and A2Sg1Ga1F were identified as the most abundant, whereas A2G0F and A2G1F dominated the Fc regions [39]. It is important to note that the composition of glycans in the Fc domain directly affects receptor binding and regulates immunity, while Fab glycosylation directly influences several functions of IgGs, including antigen binding, protein–protein interaction, antibody half-life, antibody aggregation, and immune-complex formation [38,40,41]. The aforementioned study agreed with the results obtained for the Fc region glycoforms. However, greater variability in the Fab region was observed. While many glycoforms matched between both studies, the predominant glycoforms differed, as in the present study, H-N88 residue (Fab) was characterized by the presence of A2Sg1Ga1F (24%), A1G1M4F (15%), and A1Ga1G1F (12%) as majority glycoforms. Thus, our experimental data confirmed the high structural complexity existing in cetuximab, suggesting that glycosylation in the Fab region poses a significant complexity issue for the drug. As the N-glycan levels remain unchanged under the applied stress conditions, no safety and efficacy concerns can be associated with the glycan profile when the drug is subjected to the stimuli.

### 3.7. Enzyme-Linked Immunosorbent Assay (ELISA)

MAbs can undergo physicochemical changes that can impair antigen–antibody binding when exposed to stress conditions, including some conditions related to their handling. Cetuximab biological activity was studied using an ELISA-based binding assay, which is suitable for evaluating the impact of these modifications on its EGFR binding capacity [3,13]. Here, an indirect non-competitive ELISA method adapted from our previous studies was developed and validated [3].

**Figure 7:**
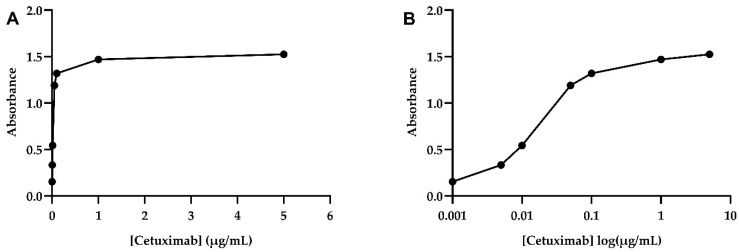
Calibration curve for cetuximab ELISA analysis. (**A**) Standard curve. (**B**) Logarithmic scale curve.

The calibration model (Figure 7) was established by selecting the mathematical function that best fits the experimental data. A logarithmic model (a = 0.3209 and b = 2.1522) was selected as the most suitable, given its R^2^ (98%) and *p*-value (0.0101), which demonstrate, respectively, the goodness of fit and the significance of the selected mathematical model (Table 6).

The method’s precision and accuracy were evaluated, and the results are expressed in terms of RSD% and R%, respectively, as set out in Table 7. According to the literature, values of ±20% are recommended as default acceptance criteria for the precision and accuracy of immunoassays [42]. Precision and accuracy assays did not exceed the recommended limit. However, the precision assay approaches 20% at the lowest concentration point (0.01 µg/mL). According to the available literature, immunoassays are studies characterized by intrinsic method variability, which becomes more pronounced as the concentration of the sample used decreases. In addition to the inherent variability associated with the technique, non-linear regression models amplify variability, particularly at very low concentrations, as observed in this case [43,44,45]. Therefore, the model was accepted, as it met the precision criteria. Thus, the ELISA method proposed proved to be suitable for the quantification of the biological activity of cetuximab.

This ELISA method was ultimately employed to determine the biological activity of the drug when subjected to different stresses. Table 8 compiles the estimated RBA% values, as well as the resulting *p*-value from the statistical analysis at each concentration point. It was assumed to have a binding capacity of 100%, for the control samples, and it was considered a positive control of degradation GnHCl, whose avidity estimation was 1%.

**Figure 8:**
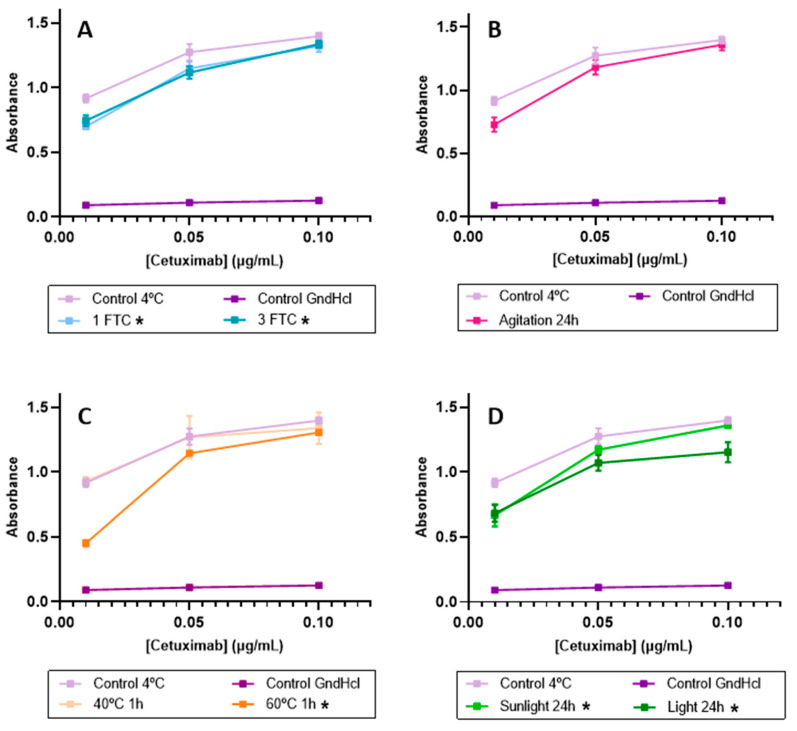
ELISA binding assay comparing control and stressed samples. (**A**) Freeze-thaw cycles stress, (**B**) agitation stress, (**C**) temperature stress, and (**D**) light stress. Conditions marked with asterisk (*) showed statistical differences when compared to the control.

Regarding FTC, RBA%s of 88% and 90% were estimated for one FTC and three FTCs, respectively. The student’s T analysis reported, in both conditions, a *p*-value < 0.05 (Table 8) for all concentrations analyzed. Considering these results, it is proposed that samples subjected to FTC experienced a reduction in EGFR binding capacity compared to the control, although it cannot be deemed a drastic decrease (Figure 8A).

A remaining activity of 91% was estimated for the agitation stress (Figure 8B). Nevertheless, significant differences were not found when applying Student’s T analysis to these conditions, as the p-values for all concentrations were >0.05. Then, no modifications to the biological activity are proposed.

For the samples subjected to heat, no changes were detected when the samples were placed at 40 °C for one hour since the RBA% was estimated as 100%, and no differences were found when a statistical analysis was applied (*p*-value > 0.05). However, the ELISA assay revealed changes in the cetuximab binding capacity when the samples were subjected to 60 °C, as observed in Figure 8C. An RBA% of 81% was estimated for this temperature, and the statistical analysis revealed significant differences with respect to the control (*p*-value < 0.05). The findings here align with results from our previous studies, where cetuximab was subjected to temperatures of 50 and 70 °C for 24 h [3]. In the mentioned study, a clear loss of biological activity was detected when cetuximab was exposed to 50 °C, along with a nearly complete loss of functionality at 70 °C. Thus, the results here obtained agree with those previously obtained, confirming that, from 50 °C, heat reduces cetuximab’s binding capacity to its therapeutic target, i.e., to the EGFR.

In reference to light irradiation, RBA%s of 89% and 72% were estimated for daylight and accelerated light expositions, respectively (Figure 8D). The statistical analysis revealed significant differences with respect to the control (*p*-value < 0.05) for the two situations tested. Thus, these results showed an incipient loss of functionality in the samples subjected to daylight, while a significant drop in the EGFR binding capacity took place when light irradiation was applied in the UV aging chamber. Again, these results confirmed those obtained in our previous studies, where a clear loss of biological activity was observed when simulating light exposure in a UV aging chamber [3].

Summarizing, the results indicated that sample exposition to FTC, 60 °C, and daylight resulted in a diminished cetuximab-EGFR binding capacity. Notably, forced light degradation exhibited a more pronounced loss of functionality, while the remaining conditions showed a mild reduction in functionality.

## 4. Discussion

Cetuximab (Erbitux^®^, 5 mg/mL) is a mAb administered in hospitals, where it may be exposed to various stress factors during compounding handling, storage, and administration processes, leading to degradation. Here, it has been checked as to whether Erbitux^®^ (5 mg/mL) undergoes physicochemical alterations under different environmental conditions that are likely to occur during handling in hospitals before administration to patients [14,15,38]. First, control samples of cetuximab were analyzed and characterized, which were then compared to samples subjected to several stress conditions. The purpose of these tests was to detect and identify potential changes in the drug product, such as aggregation, protein denaturation, structural changes, and chemical modifications in cetuximab, which could impact negatively upon cetuximab’s functionality, as evaluated by means of the binding capacity of the protein drug to its therapeutic target, the EGFR.

For cetuximab (Erbitux^®^, 5 mg/mL) control samples, the results revealed that the protein was entirely a population of natural monomers (see Table 5), confirmed by DLS and SEC. In addition, DLS estimated an HD of cetuximab monomers of around 13 nm, therefore, indicating a size slightly larger than the other IgG1 in a solution (around 10 nm) (Table 4). Similar to other mAbs [13,20], its secondary structure primarily comprises a β-sheet (42%) and unordered (33%) components (see Table 2). The main spectral DC parameters revealed a negative maximum at 218 nm, a shoulder at approximately 229 nm, and a wavelength at ellipticity 0 of roughly 209 nm. Again, these are typical values obtained for other therapeutic mAbs [12,20,21]. Cetuximab’s tertiary structure was assessed using IT-FS, revealing a C.M. of 351 nm, confirming the burial of Trp residues within the protein structure (Table 3). UHPLC-MS/MS provided information about specific structural modifications, such as the glycoform profile. In agreement with the available literature [29,32], two N-glycosylation sites in the cetuximab structure were identified, located at H-N88 and H-N299. The presence of the N-glycosylation in H-N88 provides cetuximab with a higher degree of structural complexity regarding other therapeutic mAbs in which only the conserved Fc glycosylation site is present [9]. Also, the results here proposed for the particular glycosylation pattern agreed with those previously reported [43,44], confirming the predominance of galactosylated forms rich in sialic acid in the H-N88 glycosylation site, and mainly fucosylated and rich in mannose for the H-N299 site. Chemical structural modification related to degraded processes, such as deamidation, oxidation, etc., were not detected in the cetuximab control samples, as expected.

Regarding the impact of the stresses on the N-glycosylation profile, its robustness against degradations was corroborated. N-glycosylation is a PTM necessary for protein, as it has been shown to enhance protein solubility and stability [35,38,41]. Glycan compositions also play an important role in protein functionality [46]. For example, high-mannose glycan patterns are associated with a fast clearance rate and a shorter half-life in circulation, making their absence a desirable characteristic. In the present study, only one high-mannose glycoform with a relative abundance greater than 5% (M5) was identified at the H-N299 residue (7% relative abundance), and it remained unchanged under the applied stress conditions. Core fucosilation was also detected in all the glycans identified in H-N88 and in all, except one, of the glycans in H-N299. Core fucose can decrease antibody-dependent cell-mediated cytotoxicity (ADCC) and Fc–FcγRs binding. As the stress did not affect the glycans patterns, these functions were not affected either. It is also important to note that an N-terminal pyroglutamate was detected in the structure of all cetuximab samples, control and stressed, specifically at H-Q1. This modification is not a result of PTM under stress conditions but is inherently present in the primary structure of cetuximab. The impact of N-terminal glutamine cyclization on the activity of the antibody remains unclear, although it is known that it contributes to mAbs heterogeneity [36].

To study the effects of FTC, Erbitux^®^ (5 mg/mL) was subjected to 1 FTC and 3 FTC. It is widely recognized that protein aggregation is the primary degradation pathway observed during FTC, resulting from partial denaturation during freezing [47]. However, no structural or chemical changes were reported in cetuximab when subjected to FTC, although the ELISA results revealed a slight decrease in the cetuximab-EGFR binding capacity to 90% (three FTC) and 88% (one FTC). These results suggest that the avidity of the drug is minimally affected when it is submitted to FTC, although no physicochemical changes were detected by the techniques used in this study that could justify the impact of this stressor on the EGFR-cetuximab binding. Interestingly, the cetuximab technical report does not provide specific information about the FTC. Then, based on the results shown here, although the impact is not dramatic, it is advisable to avoid freezing the medication.

Regarding agitation stress, and from a general point of view, the major degradation pathway induced by this condition is aggregation, which is highly influenced by factors such as sample pH, salts, or excipients. Therefore, controlling agitation conditions and optimizing these parameters becomes crucial to maintaining the integrity of the mAb throughout its handling in hospitals [22]. No significant changes were detected either in the physicochemical study or the functional study. Thus, it maintained a stable structure–function relationship, i.e., with no impact on the EGFR-cetuximab binding.

The heat-stress study was developed by subjecting the drug product (Erbitux^®^, 5 mg/mL) to different temperatures, 40 °C and 60 °C, over 1 h. No significant changes were observed in the primary structure, aggregation profile, or biological activity of cetuximab when subjected to 40 °C. However, exposure to 60 °C induced significant changes in the aggregation profile, with a high molecular weight species detected at 5.70 min (see Table 5), as well as in the drug avidity, which experienced a substantial reduction in its biological activity, estimating its RBA% at 81% (see Table 8). Moreover, the HD and PDI studied by DLS revealed a slight increase in both parameters compared to the control sample, indicating the appearance of larger particulates as a possible consequence of aggregate formation, which agrees with the SE/UHPLC-UV results. The CD results showed a slight variation in the spectrum of this stressed sample when compared to the control one, although the different parameters analyzed (Table 1) indicate that the secondary structure of CT is being maintained. These results are consistent with the available literature, as it is notably known that one of the primary degradation pathways resulting from high-temperature conditions is aggregation, which can lead to the loss of the native globular structure of proteins [22,48], resulting in a lesser binding capacity to the therapeutic target, as occurs with cetuximab. Therefore, these results highlight the importance of avoiding exposure of the drug to high temperatures since not only the resulting aggregation can decrease the drug avidity, thereby altering its effectiveness but it could also enhance the immunogenicity [14,15].

Light exposure is a critical environmental factor that can affect all stages of a drug product, from production to administration to patients. It is notably known that the major degradation pathway is light-induced aggregation, so it is essential to study its impact on product quality [22]. Regarding samples submitted to daylight for 24 h, a high molecular weight species was detected by SEC at 6.88 min but with a very low relative abundance of 0.80% (see Figure 3 and Table 5). Although the relative abundance of this oligomer was low, this finding suggests that sunlight promotes an incipient degradation pathway, leading to the formation of aggregates. However, DLS did not detect this oligomer, due to the very low concentration. This daylight exposure did not promote significant alterations in the secondary and tertiary structures of cetuximab. A slight increase in the oxidation of methionine residues (H-M254 and H-M430) was detected. This finding is in line with the available literature, as it is known that tryptophan, methionine, and histidine are highly susceptible sites for photo-induced degradation [22]. Then, all these slight physicochemical modifications lead to a slight decrease (around 10%, Table 8) in cetuximab’s binding capacity to the EGFR (Figure 8).

All these physicochemical changes intensified when the cetuximab samples were submitted to accelerated light in a UV aging chamber for 24 h, corroborating these pathways of cetuximab degradation leading by light, as expected. When exposed to daylight, there were no significant alterations in the secondary structure of cetuximab through CD evaluation when submitted to accelerated light irradiation. Modifications were observed when the tertiary structure of the protein was checked, not in the C.M. value—suggesting no protein unfolding in the IT-FS spectra—but just in a small decrease in the intensity, which could be attributed to the light-induced oxidation of tryptophan residues. These results were confirmed by the LC/MS(Orbitrap), detecting the incipient oxidation in H-W52 and H-W94 residues increasing up to 2.55% and 1.67%, respectively. Regarding particulates in the solutions, accelerated light exposition induced cetuximab degradation by promoting both aggregation and fragmentation, with a total of five new molecular weight species detected by SE/UHPLC-UV results (Table 5). In consequence, the relative abundance of the natural monomer decreased to 83.95%. As well as aggregation, the presence of fragmentation in the drug can affect product quality, due to it can lead to a loss of biological activity, a reduction in drug half-life, or an increase in immunogenicity due to the generation of new epitopes [22,48]. In addition, an LC-MS(Orbitrap) analysis confirmed the oxidation of the same two methionine residues oxidized by the effect of the daylight exposition but in more extension (22.23% in H-M254 and 23.78% in H-M430). These findings are in line with the available literature, which reported that, in IgG1 mAbs, these methionine residues are more susceptible to oxidation as they are located at the solvent-exposed CH2-CH3 interface. Furthermore, the oxidation of methionine can lead to significant structural changes in the IgG1 Fc fragment, particularly around the CH2-CH3 interface, resulting in a notable decrease in the avidity of mAbs for their receptor [35]. In this study, an ELISA assay confirmed that a significant detriment in cetuximab-EGFR binding occurred, estimating the drug RBA at 72%. All these findings clearly indicate that exposure to light stress, both sunlight and accelerated exposure, causes significant degradation of cetuximab, mainly due to aggregation and oxidation. Therefore, it is crucial to prevent the drug product from being exposed to light during the handling process in any context.

## 5. Conclusions

The aim of this study was to investigate whether cetuximab (Erbitux^®^, 5 mg/mL) undergoes physicochemical alterations related to its CQA under different environmental conditions, which could be related to common handling/mishandling in hospitals and could impact negatively on the antigen–antibody binding and, therefore, in the functionality. To this aim, a wide comprehensive analysis of the drug product was first performed, developing and applying a wide set of different and informative analytical techniques and strategies that led to a deep knowledge of this biotechnological formulation. Similar secondary and tertiary structures to those of other therapeutic IgGs were confirmed. Particulate in the drug product was identified as monomers with no aggregations. The primary sequence of the cetuximab was confirmed by peptide mapping obtained by LC/MS(Orbitrap), and no chemical modifications, such as deamidation and oxidation, were detected. It was also detected that there was 100% pyroglutamic acid formation in the N-terminal glutamic acid. The glycosylation patterns of the two N-glycosylation sites were identified, revealing the core fucosilation in almost all the glycans detected.

Regarding the CQAs analyzed after submitting the samples to degradation, it can be concluded that cetuximab formulated in the innovative medicinal product Erbitux^®^ (5 mg/mL) has been shown to be robust against degradation when submitted to agitation (shaking at 300 rpm for 24 h) and temperature (up to 40 °C), to which it can be exposed in hospitals when handling for administration. Exposure to the effect of light promotes protein degradation even when exposed to daylight at room temperature (excursion from 10 to 31 °C), since after 24 h, incipient aggregation was detected, which dramatically increased when the Erbitux^®^ (5 mg/mL) sample was submitted to accelerated light exposure, leading also to fragmentation. Two methionine residues were also identified as the most prone to be oxidized by light. All of this modification is promoted by the light impact on the EGFR-cetuximab binding, decreasing its functionality. Heating at 60 °C (for one hour) also promotes degradation by aggregation, decreasing the capacity of cetuximab to bind to the EGFR. Although there is no indication in the cetuximab technical report about freezing–thawing of the drug product, the results of this work indicate that it should not be frozen, since this process reduces the binding capacity of cetuximab to the EGFR, not having identified any physicochemical modification that would justify this behavior. This could be attributed to slight conformational changes in the tertiary structure that could not be detected by the proposed strategy based on IT-FS.

## Figures and Tables

**Table 1:** Cetuximab (5 mg/mL) CD spectral features: control and stressed samples (mean ± standard deviation from 3 replicates).

Stress	Wavelength (nm) (Ellipticity = 0)	Negative Maximum (nm)	Broad Shoulder (nm)
Control	208.8 ± 0.1	217.8 ± 0.1	229.4 ± 0.1
40 °C 1 h	208.7 ± 0.1	217.9 ± 0.1	229.4 ± 0.4
60 °C 1 h	208.4 ± 0.0	217.7 ± 0.1	229.1 ± 0.6
1 FTC	208.7 ± 0.1	217.7 ± 0.1	229.1 ± 0.6
3 FTC	208.4 ± 0.0	217.8 ± 0.2	229.1 ± 0.5
Control agitation 24 h	208.7 ± 0.1	217.7 ± 0.1	229.5 ± 0.1
Agitation 24 h	208.6 ± 0.2	217.7 ± 0.1	229.7 ± 0.1
Control sunlight 24 h	208.5 ± 0.1	217.7 ± 0.1	229.8 ± 0.0
Sunlight 24 h	208.5 ± 0.1	217.8 ± 0.2	229.8 ± 0.3
Accelerated light 24 h	208.5 ± 0.1	218.0 ± 0.2	230.1 ± 0.1

**Table 2:** Secondary structure content (%) estimation by Dichroweb server of cetuximab (5 mg/mL) control and stressed samples. The analysis program and dataset selected were CONTINLL and SET7 respectively.

Stress	Helix	Strand	Turns	Unordered
Control	3.4	41.6	22.3	32.6
40 °C 1 h	3.7	40.8	22.3	33.2
60 °C 1 h	4.0	40.0	22.3	33.8
1 FTC	3.9	40.6	22.6	32.8
3 FTC	3.7	41.2	22.7	32.4
Control agitation 24 h	3.2	42.2	22.2	32.5
Agitation 24 h	3.5	41.5	22.4	32.7
Control sunlight 24 h	3.6	41.1	22.4	32.8
Sunlight 24 h	3.5	41.3	22.3	32.8
Accelerated light 24 h	3.6	41.0	22.4	33.0

**Table 3:** Calculated center of spectral mass (C.M.) of cetuximab (Erbitux^®^, 5 mg/mL) control and stressed samples.

Stress	C.M. of the Fluorescence Spectrum (nm)
Control	351
40 °C 1 h	351
60 °C 1 h	351
1 FTC	351
3 FTC	351
Control agitation 24 h	351
Agitation 24 h	351
Control sunlight 24 h	351
Sunlight 24 h	351
Accelerated light 24 h	351

**Table 4:** DLS parameters of cetuximab (Erbitux^®^, 5 mg/mL) control and stressed samples.

Stress	HD (nm)	PDI
Control	12.8 ± 2.9	0.26
40 °C 1 h	12.6 ± 3.5	0.19
60 °C 1 h	**13.2 ± 5.0**	**0.33**
1 FTC	12.2 ± 3.4	0.18
3 FTC	12.6 ± 3.3	0.21
Control agitation 24 h	12.2 ± 3.2	0.17
Agitation 24 h	12.4 ± 3.1	0.19
Control sunlight 24 h	12.9 ± 2.8	0.17
Sunlight 24 h	13.0 ± 3.3	0.20
Accelerated light 24 h	12.8 ± 3.0	0.21

In bold type are highlighted those features that were significantly different from control values.

**Table 5:** Overall results of experimental retention time (RT), relative abundance (RA), and estimated molecular weight (MW) in the SEC analysis. HMWS = high molecular weight species. LMWS = light molecular weight species.

Stress	Pattern	RT (min)	RA (%)	MW (kDa)
Control	Monomer	8.01 ± 0.01	100 ± 0.00	156.36
1 FTC	Monomer	8.01 ± 0.01	100 ± 0.00	156.36
3 FTC	Monomer	8.01 ± 0.00	100 ± 0.00	156.81
Agitation 24 h	Monomer	8.01 ± 0.00	100 ± 0.00	156.81
40 °C	Monomer	8.02 ± 0.00	100 ± 0.00	155.45
60 °C	Monomer	8.01 ± 0.00	91 ± 1	156.81
	HMWS	5.70 ± 0.01	8 ± 1	1163.41
Daylight 24 h	Monomer	8.01 ± 0.00	99.21 ± 0.04	156.81
	HMWS	6.88 ± 0.03	0.80 ± 0.04	418.56
Accelerated Light 24 h	Monomer	7.97 ± 0.00	83.9 ± 0.7	162.36
	HMWS	5.68 ± 0.01	0.54 ± 0.09	1180.38
		6.30 ± 0.01	2.01 ± 0.4	690.78
		6.87 ± 0.00	10.7 ± 0.2	422.21
	LMWS	8.46 ± 0.08	2.0 ± 0.2	105.77
		9.79 ± 0.01	0.8 ± 0.1	33.31

**Table 6:** Characteristic parameters of the standard calibration curve (calibration model) and the figures of merit of the ELISA method.

Parameter	Value
Mathematical model fitted	Logarithmic
Function	Y = 2.1522 + 0.3209 × log(X)
R^2^ (%)	98.0
*p*-value	0.0101

**Table 7:** Accuracy and precision of the ELISA method developed for cetuximab (CTX) functional analysis.

CTX Concentration (µg/mL)	R%	RSD%	
		Intraday	Interday (3 Days)
0.01	93.75	16.648 ± 0.001	17.134 ± 0.001
0.05	102.58	11.856 ± 0.006	13.143 ± 0.004
0.1	88.21	8.923 ± 0.007	7.174 ± 0.006

**Table 8:** Remanent biological activity (RBA) and *p*-value of Student’s T analysis of cetuximab samples after forced degradation (^1^ *p*-value for 0.01 µg/µL. ^2^ *p*-value for 0.05 µg/µL. ^3^ *p*-value for 0.1 µg/µL). Data are shown in decreasing order of activity.

Stress	RBA%	*p*-Value ^1^	*p*-Value ^2^	*p*-Value ^3^	Significance
Control	100	-	-	-	-
40 °C	100	0.788	0.554	0.536	No
Agitation 24 h	91	0.060	0.140	0.292	No
3 FTC	90	0.039	0.013	0.010	Yes
Daylight 24 h	89	0.006	0.024	0.008	Yes
1 FTC	88	0.001	0.027	0.022	Yes
60 °C	81	4 × 10^−4^	0.076	0.011	Yes
Accelerated Light 24 h	72	0.053	0.014	0.018	Yes
GndHCl	1	0.021	7 × 10^−4^	2 × 10^−7^	-

## Data Availability

The original contributions presented in the study are included in the article/Supplementary Materials, further inquiries can be directed to the corresponding author.

## Supplementary Materials

The following supporting information can be downloaded at https://www.mdpi.com/article/10.3390/pharmaceutics16091222/s1: Table S1. Experimental data for SEC calibration. Figure S1. SEC calibration function. Figure S2. Size distribution graphs by volume of (a) cetuximab (5 mg/mL) control sample and (b) the comparison of the stressed sample at 60 °C (green) with the control sample (red). Figure S3. Coverage map obtained from the enzymatic digestion of cetuximab samples and subsequent peptide mapping analysis.
