# Supplementary material for: Comprehensive Analysis of Cetuximab Critical Quality Attributes: Impact of Handling on Antigen-Antibody Binding
Source: Pharmaceutics. 2024 Sep 19;16(9):1222. doi: 10.3390/pharmaceutics16091222 (PMC11435379; doi:10.3390/pharmaceutics16091222)
Supplement: Supplementary file 1 [file pharmaceutics-16-01222-s001.zip › pharmaceutics-3151885-supplementary.pdf]

Comprehensive Analysis of Cetuximab Critical Quality Attributes: Impact of Handling on Antigen-Antibody Binding

Alicia Torres-García[1,2], Anabel Torrente-López[2,3], Jesús Hermosilla[2,3], Amparo Hernández[3], Antonio Salmerón-García[2,4], José Cabeza[2,4], Natalia Navas[2,3]*.

1 Fundación para la Investigación Biosanitaria de Andalucía Oriental (FIBAO), Hospital Doctor Olóriz. Avda. de Madrid, 15. Pabellón de Consultas Externas 2, 2ª Planta. 18012- Granada, Spain.

2 Instituto de Investigación Biosanitaria ibs.GRANADA, Granada, Spain.

3 Analytical Chemistry Department, Science Faculty, Universidad de Granada, Avenida Fuentenueva s/n, 18071 – Granada; Spain.

4 Servicio de Farmacia Hospitalaria, Hospital Universitario San Cecilio, Avenida de la Investigación s/n, 18016 - Granada, Spain;

* Correspondence author: natalia@ugr.es

Supplementary material

Table S1. Experimental data for SEC calibration

SEC: kit calibration experimental data			
		Eje Y	Eje X
Peak	Protein[1]	Molecular weight (Da)	Retention time (min)
1	Thyroglobulin	670000	6,34
2	Globulin	**150000**	**8,02**
3	Ovalbumin	45000	9,82
4	Myoglobin	17000	10,6
5	Angiotensin II	1000	Not included in the analysis

(1) AdvanceBio SEC 300A Protein Standard, Agilent Technologies Inc., Santa Clara, CA, USA)

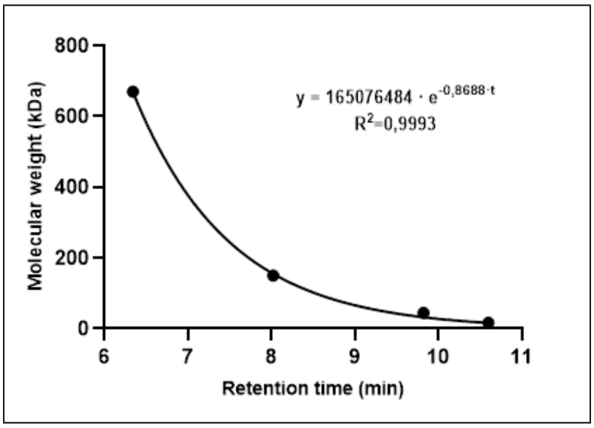

Figure S1. SEC calibration function.

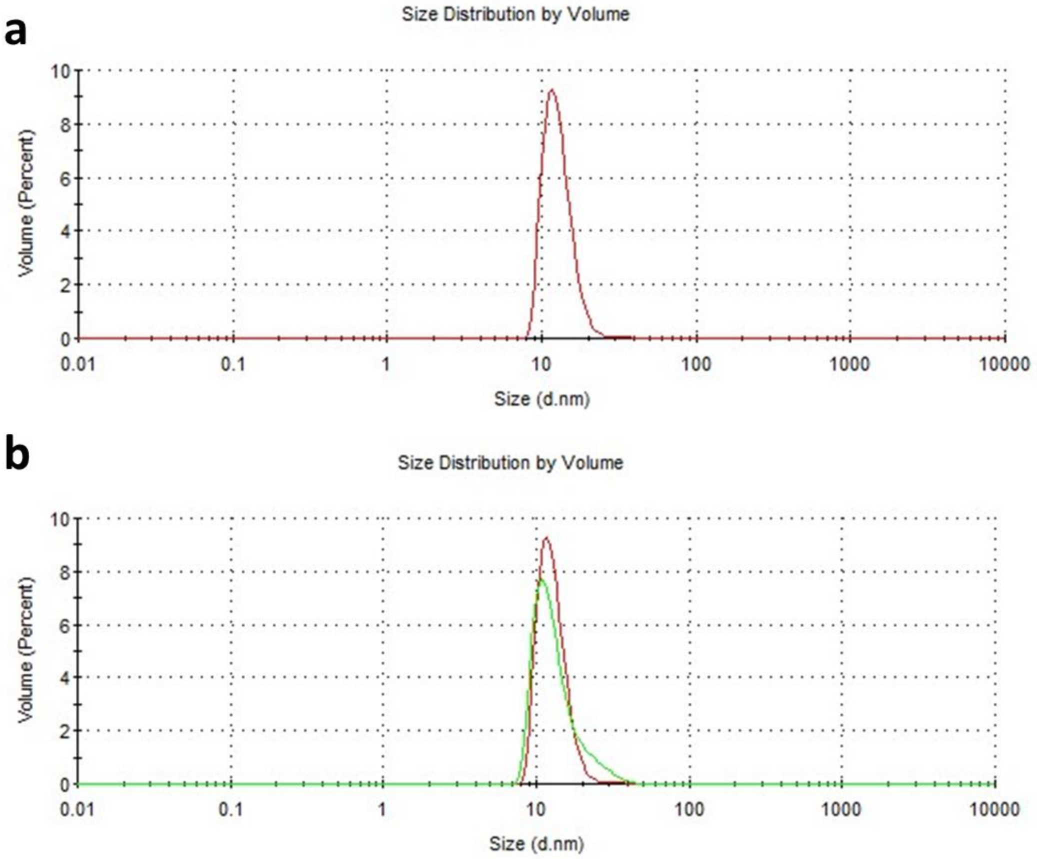

Figure S2. Size distribution graphs by volume of (a) cetuximab (5 mg/mL) control sample and (b) the comparison of the stressed sample at 60 °C (green) with the control sample (red).

Sequence Coverage Map

Created on 02/24/23
Minimum MS Signal = 174800
Data File = Ctx_1FTC_1.raw
Protease = Trypsin

Proteins	Number of MS Peaks	MS Peak Area	Sequence Coverage	Abundance (mol)
1:Cetuximab heavy chain	1044	22.7%	99.1%	39.45%
2:Cetuximab Light	612	17.6%	100.0%	60.55%
Unidentified	9812	59.8%		

Minimum Recovery = 1%
Minimum Recovery of Overlapping Peptides = 0%
Minimum Confidence = 0.9
Maximum Mass = 7000

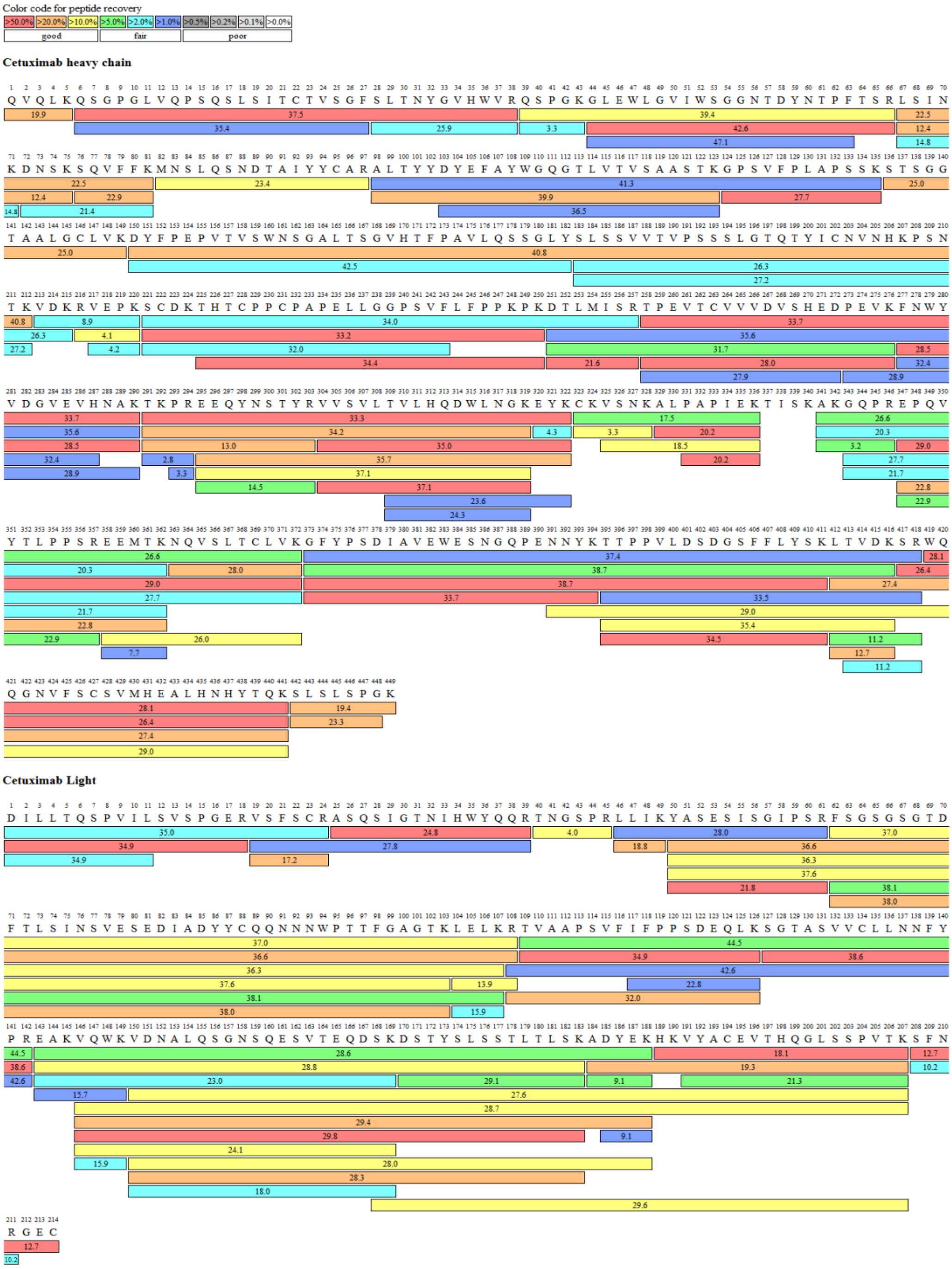

Figure S3. Coverage map obtained from the enzymatic digestion of cetuximab samples and subsequent peptide mapping analysis.
